# Effects of Dopamine on stem cells and its potential roles in the treatment of inflammatory disorders: a narrative review

**DOI:** 10.1186/s13287-023-03454-w

**Published:** 2023-08-30

**Authors:** Guan-qiao Liu, Zi-xian Liu, Ze-xin Lin, Peng Chen, Yu-chi Yan, Qing-rong Lin, Yan-jun Hu, Nan Jiang, Bin Yu

**Affiliations:** 1 Division of Orthopaedics & Traumatology, Department of Orthopaedics, Southern Medical University Nanfang Hospital, Guangzhou, 510515 China; 2 Guangdong Provincial Key Laboratory of Bone and Cartilage Regenerative Medicine, Southern Medical University Nanfang Hospital, Guangzhou, 510515 China

**Keywords:** Dopamine, Neuron stem cell, Mesenchymal stromal cell, Hematopoietic stem cell, Cancer stem cell, Inflammatory disorders

## Abstract

Inflammation is the host's protective response against harmful external stimulation that helps tissue repair and remodeling. However, excessive inflammation seriously threatens the patient's life. Due to anti-inflammatory effects, corticosteroids, immunosuppressants, and monoclonal antibodies are used to treat various inflammatory diseases, but drug resistance, non-responsiveness, and severe side effect limit their development and application. Therefore, developing other alternative therapies has become essential in anti-inflammatory therapy. In recent years, the in-depth study of stem cells has made them a promising alternative drug for the treatment of inflammatory diseases, and the function of stem cells is regulated by a variety of signals, of which dopamine signaling is one of the main influencing factors. In this review, we review the effects of dopamine on various adult stem cells (neural stem cells, mesenchymal stromal cells, hematopoietic stem cells, and cancer stem cells) and their signaling pathways, as well as the application of some critical dopamine receptor agonists/antagonists. Besides, we also review the role of various adult stem cells in inflammatory diseases and discuss the potential anti-inflammation function of dopamine receptors, which provides a new therapeutic target for regenerative medicine in inflammatory diseases.

## Introduction

Dopamine, a neurotransmitter in the human body, is vital in maintaining various physiological functions. The lack of dopamine is a crucial reason for many neurodegenerative diseases [1]. Dopamine has been applied to treat various cardiovascular diseases by increasing myocardial contractility, contraction, or relaxation of peripheral blood vessels to regulate blood pressure, thereby affecting the function of the circulatory system in the body [2–4]. In recent years, the role of dopamine in inflammatory diseases has been extensively studied, mainly focusing on the NLRP3 inflammasome, NF-κB pathway, and immune cells [5]. Meanwhile, stem cells have become important targets for treating inflammatory diseases in recent years due to their immunomodulatory function [6].

Stem cells are a group of cells with self-renewal and self-differentiation functions [7], which are classified into four categories according to their sources: ESC (embryonic stem cells), fetal and adult stem cells, and iPSC (induced pluripotent stem cells) [8]. Since adult stem cells do not cause rejection or ethical controversy, relevant studies on them have been applied in the models of various inflammatory diseases [9].

The dopamine (DA) receptor is an essential G-protein-coupled receptor (GPCR), which can be divided into two families according to its downstream signaling pathways: D1-like (D1 and D5) and D2-like (D2, D3, and D4) receptor families [10]. Several literatures have reported the expression of dopamine receptors on the surface of various adult stem cells [11–16]. They regulate behavioral, motor, and endocrine functions and are essential molecules that connect the nervous system and the immune system [5].

## Neural stem cells

### The influence of dopamine-related signaling pathway on neural stem cells

Most neural stem cells congregate in the subependymal region (SVZ) and the subgranular region of the hippocampus odontoid gyrus [17, 18]. In the presence of epidermal growth factors and fundamental fibroblast growth factors, neural stem cells grow in nerve spheres and can self-renew and differentiate into new neurons and glial cells [19]. Neural stem cells differentiate in three main directions: (1) Type A cells: neuroblasts; (2) Type B cells: astrocytes; (3) Type C cells: transient expansion cells [17]. The local microenvironment in the sub-ependymal and sub-granular regions determines the differentiation direction of neural stem cells and neural precursors. Dopamine is one of the critical factors regulating the proliferation and differentiation of neural stem cells.

Dopaminergic neurons originate from the dense region of the substantia nigra in the midbrain and project to the subependymal region, the most active region of neurogenesis in the brain, and then develop synaptic connections with stem cells and precursor cells in the subependymal region [11, 20]. Both neural stem cells and progenitors in the subependymal region express dopamine receptors, among which D2 and D3 receptors are considered important dopamine receptors that regulate the proliferation and differentiation of these nerve cells [11].

Stimulating dopaminergic nerves by using D2 receptor agonist (quinpirole) in the subependymal region can stimulate neural stem cells and increase the number of neurons, indicating that dopamine can promote the proliferation of neural stem cells after binding to D2 receptors [21]. Moreover, the ciliary neurotrophic factor (CNTF) is an endogenous regulatory component that regulates stem cell growth by dopamine and D2 receptors [22]. CNTF is produced by the subpopulation of astrocytes [23] and is negatively regulated by cAMP. Under the impairment of the central nervous system, activation of the D2 receptor rapidly reduces cAMP and increases CNTF expression. The application of a D2 receptor agonist (Quinpirole) can stimulate neural stem cells and nerve growth in CNTF^+^ mice but has no significant effect on nerve growth in CNTF^-/-^ mice, indicating that dopamine regulates the growth of neural stem cells through the D2-CNTF pathway [21]. CNTF receptor complex is expressed in neural stem cells and consists of CNTF-specific receptors, leukemia inhibitor β, and gp130. The number of neural stem cells decreased significantly in mice lacking these receptor components, which indicates that CNTF promotes the proliferation of neural stem cells and nerve growth through binding with its receptor complex [18]. Other studies indicate that CNTF is associated with activating the JAK/STAT pathway and increases the expression of Notch1, promoting the self-renewal and proliferation of precursor cells. However, the function of neural stem cells regulated through JAK/STAT and Notch1/CNTF pathways remains to be further studied [22].

Studies have also shown that dopamine activation of D2 receptors inhibits the proliferation of neural stem cells in the forebrain. Kippin et al. demonstrated that the D2 receptor antagonist (haloperidol) could promote the growth of neural stem cells [24], which contrasts with the opinion that D2 receptors promote neural stem cell proliferation. Several researchers have offered explanations for this divergence: The first and most widely accepted explanation is that it may be related to the duration of D2 receptor antagonist and agonist exposure. Long-term (14–30 days) and shorter-term exposure completely reverse the final effect, which is related to the adaptive changes of the D2 receptor induced by long-term exposure to antagonists and agonists [21]. Secondly, the injection dose, method, and detection of nerve growth will also affect the final experimental results [21].

The D3 receptor is another dopamine receptor that has been implicated in the regulation of neural stem cell proliferation and nerve growth. Studies have shown that dopamine acting on D3 receptors promotes the growth of neural stem cells in the subependymal region and the growth of the neurons in the hippocampus, striatum, and substantia nigra. Systemic use of D3 receptor agonists stimulated the proliferation of type B and C cells, but type A cells were more significantly reduced in the absence of D3 receptors than in the absence of B and C cells. Lao and his team demonstrated [17, 25, 26] that D3 receptor activation accelerates the cell cycle of neural stem cells by activating the Akt and ERK1/2 signaling pathways, especially the Akt pathway, and thus increases the cell population [17]. Under conditions of nerve impairment, such as Parkinson's disease, activation of D3R supports the survival of neural precursor cells through the Cdk5/p35 signaling pathway [27, 28]. However, similar to the study of D2 receptors, it is controversial whether D3 receptors promote the growth of neural stem cells. Studies have shown that D3 receptors and D3 agonists have no significant effect on the proliferation and differentiation of neural precursor cells. Some researchers have attributed it to different species [29–31], but the specific reasons remain to be further studied.

### Potential role of neural stem cells in inflammatory diseases

Dopamine is not the only factor that regulates neural stem cells. Acetylcholine, norepinephrine, and serotonin regulate the proliferation and differentiation of neural stem cells. The proliferation and differentiation of neural stem cells play an essential role in different diseases. Recent studies have found that neural stem cells can be used as a new therapeutic method for some inflammatory diseases of the nervous system.

First, neural stem cells can reduce the burden of inflammation at the impairment site and reduce the number of mononuclear macrophages in the inflammatory environment. In multiple sclerosis, mononuclear macrophages appear around the white matter, activating the production of microglial nodules and T cells, leading to further inflammation and irreversible pathological changes in neurons. It suggests that the inflammatory environment mediated by mononuclear macrophages aggravates multiple sclerosis, and neural stem cells can reverse the pro-inflammatory phenotype of macrophages(M1) to an anti-inflammatory phenotype by regulating mononuclear macrophages (M2), thus alleviating inflammation and reducing inflammatory impairment in central nerve system [31].

Second, neural stem cells can secrete extracellular vesicles (E.V.s), which contain cellular proteins, lipids, and microRNAs. E.V. has low immunogenicity, and biodegradability and can encapsulate endogenous bioactive molecules and crosses the blood–brain barrier. ReN cells are a kind of neural stem cells originating from the ventral midbrain region of the human brain. The E.V.s secreted by ReN cells are rich in seven miRNAs, which can inhibit the activation of the MAPK inflammatory pathway and thus relieve the inflammation caused by stroke-induced ischemia [32]. In addition, nerve inflammation can further impair neurons in spinal cord injury and cause irreversible nerve impairment. Small extracellular vesicles (sEVs) secreted by neural stem cells can reduce NO and inflammatory factors produced by LPS-stimulated microglia, inhibit neuroinflammation and avoid secondary injury in neurons. Therefore, neural stem cell transplantation has attracted massive attention in treating spinal cord injury in recent years. Meanwhile, more and more researchers are trying to use neural stem cells on other inflammatory diseases [32], which gives stem cell therapy broad prospects in treating inflammatory diseases.

## Mesenchymal stromal cells

### Influence of dopamine-related pathway signals on mesenchymal stromal cells

Mesenchymal stromal cells (MSCs) have pluripotent differentiation ability in vitro [33], they can be isolated from various tissues, such as bone marrow, endometrium, umbilical cord, and adipose tissue [34, 35]. Under specific conditions in vitro, mesenchymal stromal cells can be differentiated into various lineages of endoderm, mesoderm, and ectoderm, such as bone, chondrocytes, adipose, muscle, neuron, islet cells, and hepatocytes [36, 37]. Although many studies have defined them as stem cells [38–40], it is rigorous to define them as stromal cells instead of stem cells because MSCs isolated in vitro are not homogeneous which contain not only stem cells, but also progenitors and differentiated cells [41, 42]. In addition, recent studies have shown that dopamine affects mesenchymal stromal cells' migration, mobilization, proliferation, and differentiation.

#### Migration mobilization

MSCs play an important role in wound angiogenesis and healing. It can be recruited to the injured site and differentiate into endothelial cells [43, 44] and release various pro-angiogenic factors (VEGF) to support the growth, survival, and differentiation of endothelial cells [45–48]. Recent studies have shown that physiological concentrations of dopamine in the synaptic cleft significantly inhibit neovascularization in wound tissue, which due to dopamine (1 μM), inhibited VEGF-induced MSCs migration by regulating the phosphorylation of VEGFR-2 and Akt and actin polymerization through mesenchymal cell surface D2 receptors [49]. Treatment with the specific D2 receptor inhibitor (itopride) significantly increased the number of MSCs in the mice's peripheral circulation. It significantly accelerated the mobilization toward the wound of exogenous MSCs and their infiltration into blood vessels, thus promoting the progress of angiogenesis [49]. However, Isabel et al. found that high dopamine (50 μM) concentration increased Akt phosphorylation and hMPCs migration by activating D2 receptors on the surface of hMPCs (human-derived mesenchymal progenitor cells) in vitro and dopamine (50 mg/kg, peritoneal injection) also enhanced mobilization of mMPCs (murine-derived mesenchymal progenitor cells) via D2 receptors [50]. This suggests that dopamine may have opposite effects on the mobilization and migration ability of mesenchymal stromal cells under different concentrations, which provides a new aspect for MSCs in the clinical treatment of vascular injury and other traumatic incidents.

#### Osteogenic and adipogenic differentiation

Bone remodeling is a dynamic process between osteoblast-mediated bone formation and osteoclast-mediated bone resorption [51]. Bone marrow-derived mesenchymal stromal cells (BMSCs) have the potential for multi-directional differentiation of osteogenesis, chondrogenesis, and adipogenesis [52], which play a crucial role in bone remodeling. Therefore, it is of great clinical value to study the mechanism regulating the function of BMSCs. Recent clinical studies have shown that compared with healthy controls, patients with Parkinson's disease have a higher prevalence of osteoporosis [53–55]. These findings suggest that dopamine may play a crucial role in bone homeostasis. Previous studies have shown that hBMSCs (human bone marrow mesenchymal stromal cells) highly express D1 and D2 receptors. In terms of proliferation, dopamine concentration has different effects on the proliferation of hBMSCs. There is no significant effect in the nanomolar range, but when the concentration reached to micromole, the proliferation of hBMSCs was promoted, especially at the concentration of 50 μM. However, the proliferation was significantly inhibited at the concentration of 500 μM [12]. In osteogenic differentiation, Zhang et al. found that dopamine (100 μM) significantly enhanced calcium signal transduction in BMSC, inhibited CREB activity, and significantly reduced CAMP-induced calcium signal transduction in BMSC [56]. As Ca^2+^ signaling is an essential factor that regulates differentiation [57], dopamine may affect the osteogenic differentiation of BMSC by regulating the cAMP-CREB pathway. Subsequently, Wang et al. found that low concentrations of dopamine (5 nM) enhance the phosphorylation of ERK1/2 by activating the D1 receptor, thereby increasing the transcriptional activity of Runx2 and promoting the osteogenic differentiation of hBMSCs. When the dopamine concentration was higher than 5 μM, it inhibited the osteogenic activity of hBMSCs [12]. A recent study by Hong and colleagues supported these findings. They found that dopamine did not interfere with the apoptosis and proliferation of rBMSCs (rat bone marrow mesenchymal stromal cells) at a concentration of 10 μM, but inhibited osteogenic differentiation of rBMSCs through the AKT/GSK-3β/β-Catenin signaling pathway, reduced ALP activity and minimized nodule formation, and decreased expression of osteogenic associated genes (*Col1a1, ALP, Runx2, Opn, and Ocn*) [58]. These studies indicated that different dopamine concentrations might cause different results in  osteogenic differentiation of BMSC.

Dopamine has been reported to have a higher affinity for D2 receptors than D1 receptors [59, 60] and will have different effects due to the opposite cAMP-modulating ability of D1 and D2 receptors. In addition, it may be because different cells may have different amounts of dopamine receptors and respond differently to dopamine at the same concentration [61]. Furthermore, dopamine promotes adipogenic differentiation of rBMSCs at 10 μM dopamine stimulation, but the mechanism needs to be further studied [61]. In summary, different dopamine concentrations are a vital factor affecting the differentiation function of bone marrow mesenchymal stromal cells and may affect their therapeutic potential in diseases such as osteoporosis and inflammatory bone loss.

### Potential role of mesenchymal stromal cells in inflammatory diseases

In recent years, mesenchymal stromal cells have received widespread attention in clinical fields, especially in inflammation. They have been proven to play an essential role in a variety of inflammatory diseases, such as graft-versus-host disease (GVHD), systemic lupus erythematosus (SLE), type I diabetes mellitus, multiple sclerosis, kidney injury, fibrosis and osteoarthritis [62–65].

Mesenchymal stromal cells alleviate inflammation and promote tissue repair through the following aspects: First, MSCs secrete multiple factors in the inflammatory environment, including anti-inflammatory mediators (PGE2, TSG6, HO1, galactolectin) [66], growth factors (HGF and LIF) [67, 68], cytokines (TGF-β) [69, 70], and extracellular vesicles [71]. These factors can inhibit the proliferation and function of pro-inflammatory immune cells and increase the number of anti-inflammatory immune cells, further inhibiting the activity and function of pro-inflammatory immune cells and promoting tissue repair [72]. Second, MSCs produce a variety of chemokines in the inflammatory environment, which can recruit T cells near MSCs [62]. Meanwhile, the inflammatory environment can also induce the high expression of nitric oxide synthase (iNOS) and indoleamine 2,3-dioxygenase (IDO) in MSCs, which can inhibit the proliferation and activity of surrounding T cells and reduce inflammatory response [73]. Third, MSCs in inflammatory states can be attacked by components of the complement system, activated neutrophils, and perforin-positive toxic cells and induce apoptosis [74]. Then, apoptotic mesenchymal stromal cells can be absorbed by phagocytes and induce the expression of IDO, which suppresses immune response [75].

Although there have been no reports of dopamine combined with mesenchymal stromal cells in treating inflammatory diseases, relevant studies have confirmed that dopamine can promote the proliferation of MSCs and affect the recruitment at the injured site after vascular trauma. This demonstrates that dopamine and its corresponding receptor inhibitors may play an essential role in inflammatory disease and regenerative medicine therapy in the future.

## Hematopoietic stem cells

### The influence of dopamine-related pathway signals on hematopoietic stem cells

Hematopoietic stem cells (also known as long-term hematopoietic stem cells, or LT-HSCs) are pluripotent stem cells with the ability to self-renewal and multiple differentiation potentials. LT-HSCs produce all blood and immune system cells in the body. LT-HSCs can differentiate into two kinds of cells, called﻿ short-term hematopoietic stem cells (ST-HSCs) and multipotent progenitor cells (MPPs), with limited differentiation potential and loss of self-renewal ability. LT-HSCs, ST-HSCs, and MPPs are collectively referred to as hematopoietic stem and progenitor cells (HSPCs) [76].

Previous analysis of RNA-seq in bone marrow showed that HSPCs expressed multiple dopamine receptors [13]. These results suggest that dopamine may directly act on HSPCs and affect their function. Recently, Liu Y et al. established a mouse model of dopamine D2 and D3 receptor deletion and found that deletion of Drd2 (Drd2^−/−^) or Drd3(Drd3^−/−^) alone or together could significantly reduce the number of HSPCs. These results suggest that D2 or D3 receptors may affect the survival of HPSCs. Then, they constructed Th-CRE/Rosa26-iDTR (DTRiΔTh) mice and injected diphtheria toxin (DTX) to block dopamine synthesis in the central nervous system. They found that HPSCs were significantly reduced in DTRiΔTh mice treated with DTX. In addition, they interfered with peripheral dopamine synthesis using carbidopa and found that HPSCs were significantly decreased in mice treated with carbidopa. It demonstrates that dopamine can directly affect the survival of HSPCs by binding to D2-type receptors. This effect probably was due to the inhibition of cyclic adenosine phosphate (cAMP)/protein kinase A (PKA) signaling pathway by binding to D2-type receptors, which led to the up-regulation of Lck protein (a member of the Src family of kinases). Further, it influenced the phosphorylation of c-kit and activation of ERK [77]. In addition, Ankita Kapoor et al. also found that dopamine promoted the growth and differentiation of hematopoietic stem cells (HPCs) by investigating the growth and development of lymph glands in Drosophila flies [78].

Agarwala et al. [79] recently analyzed the ultrastructural of HSPCs in the zebrafish larval kidney marrow (K.M.) niche using two correlative light (CLEM) techniques. Dopamine beta-hydroxylase (DBH) positive sympathetic ganglion cells were directly adjacent to HSPCs, and inhibition of DBH significantly reduced the number of HPSCs. This finding suggests that DBH-positive cells may be in direct contact with HSPCs and significantly impact the function of HSPCs. DBH is a mono-oxygen enzyme that converts dopamine into norepinephrine [80], suggesting that dopamine may indirectly affect the viability of HSPCs by converting them into norepinephrine.

In recent years, with deep research on the function of dopamine, the relationship between Dopamine and HSPCs has attracted more attention. Dopamine's direct and indirect effect on HPSCs may provide a theoretical basis for its future application in various hematopoietic systems, immune systems, and inflammatory diseases.

### Relationship between hematopoietic stem cells and progenitor cells (HSPCs) and inflammation

Typically, the number of HSCs in the bone marrow is minimal, and most are inactivated. HSCs are activated only when other hematopoietic or immune system cells are significantly reduced for various reasons (inflammation, infection, and acute blood loss). Once activated, HSCs can generate a daughter cell with kept HSC potential and a hematopoietic progenitor cell (HPCs) through asymmetric division [81, 82]. HPCs entering the myeloid differentiation pathway are called myeloid progenitors (CMPs), which can differentiate into myeloid cells (erythrocytes, megakaryocytes, monocytes, and neutrophils). While HPCs entering into the lymphoid differentiation pathway are called lymphoid progenitors (CLPs), which can differentiate into lymphoid cells (such as T and B lymphocytes and natural killer cells) [83, 84]. Mononuclear macrophages, granulocytes, N.K. cells, and other cells are important components of innate immunity, while T and B lymphocytes are essential components of adaptive immunity. Innate immunity and adaptive immunity constitute the immune response system in the human body, which eliminates pathogens and aging, dead cells, and other "non-self" substances.

Inflammation is a physiological response to various stressors, such as infection and trauma, which trigger protective immune responses involving immune cells, blood vessels, and various cytokines [85]. Compared with secondary lymphoid organs, which are mainly responsible for immune activation, primary lymphoid organs are commonly considered to be waived by immunity and less exposure to immunogenic substances, so the traditional concept considers HSPCs to play little role in inflammatory damage. However, recent studies have shown that acute or chronic inflammation in the body can directly stimulate HSPCs and thus affect the various processes of its growth and differentiation. For example, sepsis promotes the myeloid differentiation of HSPCs in the bone marrow and peripheral blood to produce many neutrophils. This adaptive response of HSPCs to inflammation can promote the elimination of inflammation [76, 86, 87], indicating that HSPCs play a vital role in the body's response to inflammation.

Toll-like receptors (TLRs) can recognize the microorganism antigen of bacteria or viruses and initiate the innate immune response. Nagai et al. found that a variety of TLRs existed on the surface of HSPCs, and the activation of TLRs could transform HSPCs from inactivated to an active state [88]. Subsequent studies have further demonstrated that inflammatory states can affect the function of HPSCs by activating TLRs. For example, systemic administration of LPS (a component of the Gram-negative bacterial outer wall) could enhance the proliferation ability of HSCs by activating TLR4 receptors and be more inclined to myeloid differentiation to cope with the body's inflammatory state [89, 90]. In addition, various growth factors and cytokines produced in inflammatory states (such as G-MCF [86, 91], IL-1 [92], and IFNs) can also affect the function of HSPCs in the bone marrow and peripheral blood.

In conclusion, inflammation can lead to the proliferation and differentiation of HSPCs, which can eliminate inflammation. The interaction between HSPCs and inflammation indicates that HSPCs play a significant role in coping with inflammation.

### HSPCs may be a potential target for dopamine to relieve inflammation

The ability of HSPCs to sense inflammatory stimulation and make adaptive responses plays a crucial role in acute inflammation or infection. Various inflammatory cytokines during infection or inflammation enhance the proliferation and differentiation of HSPCs, but persistent chronic inflammation may also lead to the failure and dysfunction of HSPCs [76]. In recent years, many articles have shown that dopamine plays a crucial role in maintaining the physiological activity of HSPCs. Although there are no reports on the role of dopamine and HSPCs in treating inflammatory diseases, its effect on the function of HSPCs may be one of the potential targets for alleviating various inflammatory diseases.

## Cancer stem cells

### The influence of dopamine-related pathway signals on cancer stem cells

Cancer stem cells, as a small subgroup of tumor cells [93], can self-renewal, generating heterogeneous cancer cells and maintaining tumor growth [94]. Circulating, disseminated, and metastatic initiation cancer cells are all derived from cancer stem cells [95]. Cancer stem cells are one of the leading causes of cancer growth, metastasis, and drug resistance [96] and are the only tumor cells capable of infinite growth and metastasis [97]. The Hedgehog, Notch and Wnt pathways are related to cancer stem cells, and blocking these pathways inhibits the growth activities of cancer stem cells [98]. Traditional antineoplastic drugs primarily target differentiated cancer cells [99]. However, because cancer stem cells often stagnate in the G0 phase of the cell cycle with low proliferation capacity, traditional antineoplastic drugs show little effect on cancer stem cells. Such as 5-FU and cisplatin, two traditional antineoplastic drugs, have been resisted by many lung cancer patients, resulting in less efficacy in lung cancer therapy [98]. Moreover, most of these drugs lack specificity, so they can also produce toxic effects on normal human cells [99].

Dopamine and its receptors are important factors in regulating cancer stem cells. A study has shown that dopamine can reduce the frequency of cancer stem cells and induce apoptosis of cancer stem cells in vitro in breast cancer [100], and the signaling pathway of dopamine receptors is the only drug-receptor pathway that has been found to have specific effects on cancer stem cells [101]. It makes dopamine-related drugs a new therapeutic strategy for cancer. The mechanisms by which dopamine regulates cancer stem cells are broadly divided into two categories: inhibiting the proliferation and inducing death of cancer stem cells, and promoting the differentiation of cancer stem cells into non-cancer cells, thereby reducing drug resistance and reducing cancer recurrence. Due to the specificity of tumor tissues, dopamine receptor types, and distribution, dopamine receptor agonists and antagonists have different antineoplastic effects in different tumor tissues.

D1 receptor agonists have antineoplastic effects on breast cancer [99]. D1 receptor agonists fenoldopam (FEN) and L-SPD significantly inhibited breast cancer stem cells in traditional antineoplastic drug-resistant breast cancer, and FEN and L-SPD had sustained inhibition effects on breast cancer stem cells after short exposure. In drug-resistant breast cancer, one dose of dopamine can inhibit cancer stem cell proliferation for 72 h, and one-week treatment of FEN can inhibit breast tumor proliferation for two weeks [95, 102].

In pituitary adenomas, D2 receptor agonists are the key that inhibits tumor growth. In physiological states, D2 is responsible for regulating the effect of hypothalamic dopamine on different pituitary cells and inhibiting the secretion and synthesis of pituitary PRL. Clinical trial results have shown that the D2 receptor was highly expressed on the surface of pituitary adenoma cells and its stem cells in patients with pituitary adenoma with a significant secretion of PRL [14, 15]. The combined application of somatostatin analog, DA, or D2 receptor agonist effectively reduce the secretion of a large amount of PRL by pituitary adenoma cells through physiological feedback mechanisms and inhibit the growth of tumors. Moreover, the D2 receptor agonist plays an antimitotic role in pituitary adenoma stem cells, significantly reducing the proliferation rate and activity and playing an antineoplastic function [14]. However, studies have proved that there is a kind of pellet cell with the characteristics of pituitary adenoma stem cells in the rat pituitary adenoma. D2R was a low expression, but CD133 was high on the surface of these cells, making them insensitive to D2 receptor agonists.

Moreover, after treatment with a D2 receptor agonist, D2 receptor expression on the surface of these cells gradually decreased, while CD133 expression gradually increased. The possible reason for this phenomenon is the methylation of the D2 receptor DNA promoter. However, this conclusion remains to be further verified [16]. Tumor recurrence after drug withdrawal is a significant problem in treating pituitary adenoma, and low expression of D2 receptors in pellet cells may be the reason.

However, DA receptor antagonists also show antineoplastic function in some cancer stem cells. Methoprazine, a D2 receptor antagonist, has been shown to have a more specific inhibitory effect on human lung cancer stem cells, colorectal cancer stem cells, breast cancer stem cells, glioblastoma cancer stem cells, and T-lymphocytic leukemia by inhibiting proliferation, inducing apoptosis and differentiation in tumor stem cells [16]. Methoprazine can target the formation of lung cancer stem cell spheres, inhibit their proliferation, and directly induce the death of human lung cancer stem cells under high-concentration dosage. Meanwhile, Methoprazine inhibited the phosphorylation of Akt, an important protein that maintains the properties of cancer stem cells and causes stem cells to lose their stem cell character. Methoprazine can also inhibit the proliferation and migration of hepatocellular cancer stem cells by inhibiting stem cell characteristics associated with genes such as CD133, EpCAM, OCT4, SOX2, KLF4, and MYC. When the expression levels of these genes are down-regulated, cell proliferation is also reduced [104]. Therefore, combining Methoprazine with traditional antineoplastic drugs 5-FU and cisplatin can play a more effective antineoplastic effect in cancer therapy [98].

Though D2 receptor antagonists have been shown to inhibit the proliferation of glioblastoma neural stem cells (GNS) [98], several clinical trials results have shown that D4 receptor antagonists are more specific to glioblastoma neural stem cells than D2 receptor antagonists in vivo. D4 receptor antagonists inhibit cellular autophagolysosomal degradation, alter lipid/cholesterol synthesis, and lead to many intracellular autophagic vacuoles and cholesterol accumulation. Moreover, after the inhibition of the D4 receptor, the expression of the GNS-specific molecule PDGFRβ and its downstream effector ERK1/2 are decreased, which finally inhibited GNS self-renewal and tumor growth [103, 104].

What is more, as a D2 receptor partial agonist, aripiprazole stabilizes the activation level of the DRD2 downstream signaling pathway through competitive binding. It can not only inhibit the growth of cancer stem cells and induce apoptosis of cancer stem cells but also promote the differentiation of cancer stem cells into non-cancerous cells [101, 105]. It has been proven that aripiprazole down-regulates the proportion of cancer stem cells in tumor cells by inhibiting the Wnt/GSK3β/β-catenin pathway. After binding to receptors on the cell surface, GSK-3β is phosphorylated, and β-catenin migrates into the nucleus, interacting with T cell-specific factor (TCF) or lymphoid-enhanced binding factor (LEF) to promote the expression of downstream genes [106]. On the other hand, aripiprazole interferes with the localization of β-catenin in the nucleus, thereby affecting the Wnt/β-catenin signaling pathway [14] [105]. Conversely, another study showed that aripiprazole promotes the phosphorylation of GSK-3β [107]. We suspect this bias may be related to the source and species specificity, D2 receptor activity, and initial activation of Wnt/GSK3β/β-catenin signaling pathway in the tumor stem cells'. Survivin is an anti-apoptotic protein highly expressed on the surface of cancer stem cells, making tumors resistant to multiple antineoplastic drugs and inhibiting the human body's immune response to tumors. Aripiprazole reduces the expression of surviving intracellular levels, which can promote apoptosis in cancer stem cells [107, 108].

### The clinical therapeutic prospect of the dopamine signaling pathway in cancer

Dopamine and its receptor agonists/antagonists have antineoplastic effects and can enhance the effect of other antineoplastic drugs. Sunitinib is a multi-targeted antineoplastic drug that can inhibit breast cancer growth by inhibiting tumor angiogenesis. Still, it may also activate the Notch signaling pathway, which stimulates the generation of cancer stem cells [109]. The combination of dopamine and sunitinib can significantly enhance the efficacy of sunitinib and inhibit the tumor growth and the cancer stem cells generation, thus reducing the drug resistance of tumors and the chance of tumor recurrence [99].

## Future perspectives

Many studies have shown that dopamine receptors are widely present in the cell membranes of neural stem cells, mesenchymal stromal cells, hematopoietic stem cells, and cancer stem cells. Dopamine activates CNTF, Akt, and ERK1/2 signaling pathways by binding with D2/D3 receptors to promote the proliferation and differentiation of neural stem cells. It regulates the phosphorylation of VEGFR-2 and Akt, actin polymerization, and calcium signaling by binding to D1/D2 receptors and influences the mobilization, migration, and osteogenic differentiation of mesenchymal stromal cells. Researchers have recently found that dopamine plays a vital role in maintaining hematopoietic stem cell proliferation and differentiation. Although the underlying mechanism of action is still unclear, the absence of type D2 dopamine receptors and the dysfunction of dopamine synthesis in vivo can significantly reduce the number of hematopoietic stem cells. In addition, dopamine, as the only substance that has specific effects on cancer stem cells, can significantly inhibit the proliferation and differentiation of cancer stem cells, which makes dopamine and its related drugs become potential targets for cancer treatment (Table 1).

**Table 1:** Role of dopamine and its receptor on mesenchymal stromal cells and neuron, hematopoietic, cancer stem cells

Cell type	Classification	Receptors	Signaling pathway	Outcomes
Neural stem cells		D2	D2R(+)-cAMP(−)-CNTF(+)-JAK/STAT/Notch1(+)	Promotion of the proliferation and differentiation of neural stem cells
		D3	D3R(+)-AKT/ERK1/2(+)	
		D3	D3R(+)-Cdk5/p35(+)	Protection against the loss of nestin expression caused by various stressors to reduce neural stem cell death
Mesenchymal Stromal cells (MSCs)	Human-derived mesenchymal progenitor cells (hMPCs)	D2	D2R(+)-AKT(+)	Increased mobilization and migration capacity of hMPCs
	Murine-derived mesenchymal progenitor cells (mMPCs)	D2	D2R(+)-VEGFR-2/AKT-VEGF(−)	Reduction of the number of mMSCs and inhibited their ability to mobilization and migration
			D2R(+)-AKT(+)	Increased mobilization and migration capacity of mMPCs
	Human bone marrow mesenchymal stromal cells (hBMSCs)	D1/D2	Unknown	A low concentration of dopamine (50 μM) can promote the proliferation and differentiation of hBMSCs, while the high concentration of dopamine (500 μM) can inhibit
		D1	D1R(+)-ERK1/2(+)-RUNX2	Low concentrations of dopamine (below 5 nM) can promote osteogenic differentiation of hBMSCs, while high concentrations (above 5 μM) can inhibit
	Rat bone marrow mesenchymal stromal cells (rBMSCs)	Unknown	cAMP-CREB-Ca^2+^	Affects osteogenic differentiation of rBMSCs
			AKT/GSK-3β/β-Catenin	Reduction of osteogenic differentiation of rBMSCs and promoted their lipogenic differentiation
Hematopoietic stem and progenitor cells (HSPCs)		D2/D3	D2R/D3R(+)-cAMP(+)-PKA(+)-LCK(+)-c-kit/ERK(+)	Regulation of HSPCs maintenance and enhance their ability to proliferate and differentiate
Cancer stem cells		D2	D2R(−)-Wnt/β-catenin(−)	D2 receptor antagonists aripiprazole and methoprazine can inhibit the growth of cancer stem cells and induce apoptosis
			D2R(−)-AKT(−)	While promoting the differentiation of cancer stem cells into non-cancer cells
	Pituitary adenoma stem cells	D2	D2R(+)-PRL(−)	Reduction of the proliferation rate and activity of pituitary tumor stem cells
	Glioblastoma neural stem cells (GNS)	D4	D4R(−)-PDGFRβ(−)-ERK1/2(−)	Inhibition of GNS self-renewal and glioblastoma growth
	Breast cancer stem cells	D1	D1R(+)	D1 receptor agonists fenoldopam and L-SPD can inhibit the growth of mouse breast cancer stem cells

L-SPD: L-Stepholidine

In addition, recent studies have shown that neural stem cells, mesenchymal stromal cells, and hematopoietic stem cells also play an essential role in inflammatory diseases: (1) Neural stem cells can regulate the phenotype of mononuclear macrophages or secrete extracellular vesicles to inhibit the production of various pro-inflammatory factors, thus relieving neuroinflammation; (2) Mesenchymal stromal cells can inhibit the activity of inflammatory cells by releasing various cytokines, to relieve inflammation; (3) Hematopoietic stem cells can generate a variety of immune cells through the reactive proliferation and differentiation of inflammation to eliminate stressors such as infection, thus reducing the inflammatory state of the body.

Senescence, in which cells are subjected to excessive DNA damage, oxidative stress and other harmful stimuli, causes cell cycle arrest [110, 111] and SASP secretion contained various inflammatory cytokines, proteases and lipids [112], including IL-6, IL-8, membrane cofactor proteins (MCP), macrophage inflammatory proteins (MIPs), etc. [113]. Thus, the chronic low-level inflammation associated with the aging SASP phenotype is called "inflammaging" [114]. During aging, cells continuously encounter endogenous and exogenous stress, resulting in senescent cell accumulation, especially senescent stem cells. A large number of senescent cells secreted SASP and affect surrounding normal cells, which forces the organism to be in a stage of chronic inflammation and affects normal physiological function [115]. This state of chronic inflammation that correlates with stem cell dysfunction in aging is a vital risk factor for many age-related diseases, including Parkinson’s disease, osteoarthritis, atherosclerosis, etc. [116–118].

In addition to being an important neurotransmitter, dopamine not only plays a crucial role in movement, learning and memory, etc., but also closely related to senescence: [1] Dopamine regulates the oxidative stress and chronic inflammation of the nervous system through the renin-angiotensin system (RAS) to prevent too many normal cells transform into senescence cells [119, 120]; (2) Dopamine inhibit the activation of NF-κB signaling pathway which is a major signaling pathway of SASP secretion [112, 121, 122]. Recent studies have also shown that metformin and other drugs inhibit the activation of NF-κB and SASP gene in senescence cells [123]. Therefore, dopamine may regulate the production of SASP by inhibiting the NF-κB signaling pathway; however, another study shows that dopamine undergoes autooxidation and enzyme-catalyzed reactions during senescence, causing reactive oxygen species (ROS) and toxic quinones production. The accumulation of these metabolites leads to the abnormal increase in senescence cells [124].

Dopamine has a potential role in the regulation of stem cell senescence. Although the oxidative metabolites of dopamine promote the occurrence of cellular senescence, they still relieve oxidative stress and chronic inflammation and reduce the production of SASP through various ways and delay cell senescence. Although the current study on the relationship between dopamine and senescence is unclear, much evidence indicates that dopamine may positively affect senescent cells. Identifying the relationship between dopamine and senescent cells may provide a new therapeutic strategy for various age-related diseases.

## Conclusions

**Fig. 1:**
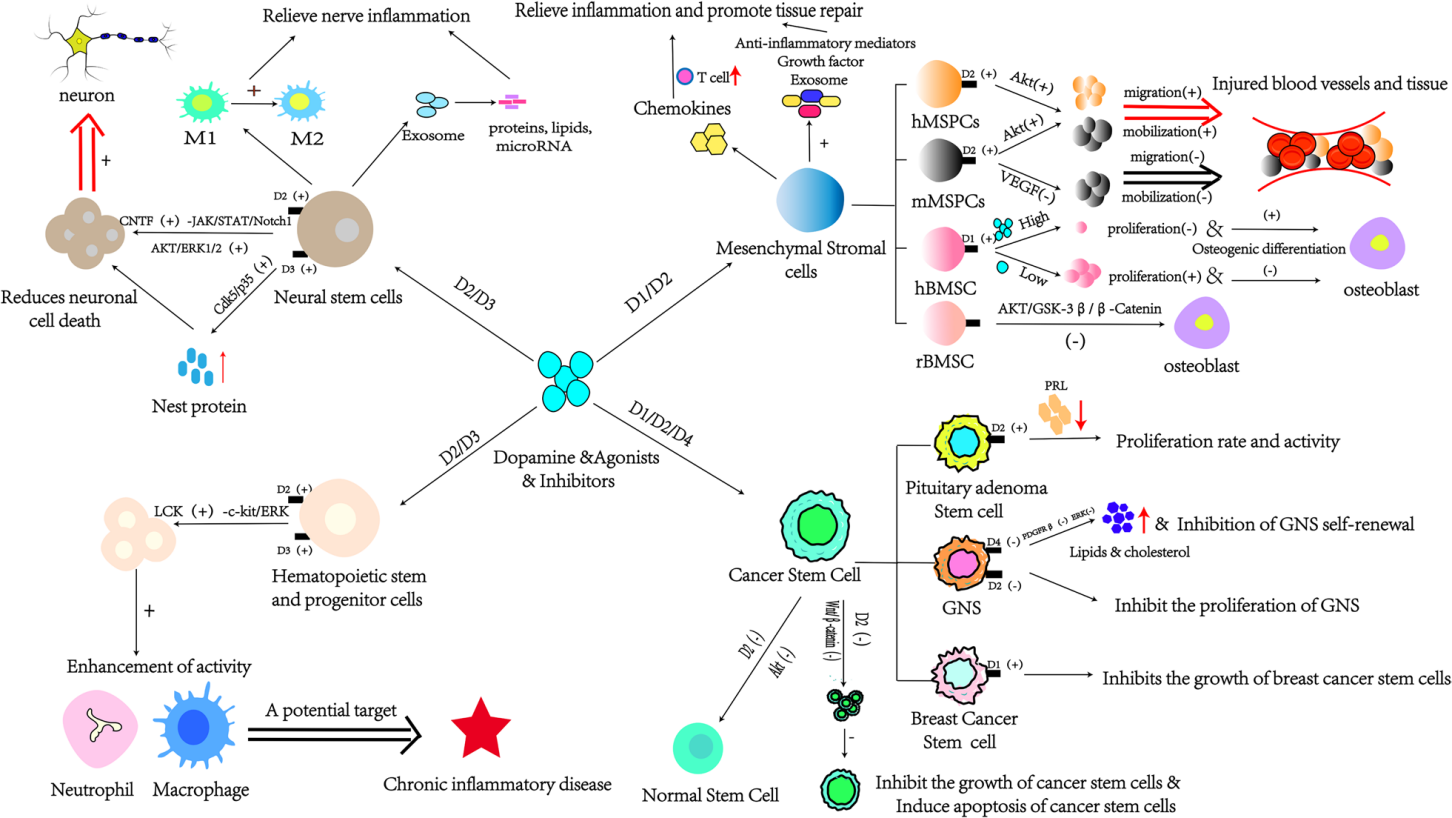
Mechanism of how dopamine and its receptor regulate mesenchymal stromal cells and neuron, hematopoietic, cancer stem cells (This image is depicted by our team)

In conclusion, dopamine affects various functions of stem cells by binding with dopamine receptors, and stem cells make adaptive responses to the inflammatory state of the body and, in turn, affect the occurrence of inflammation (Fig. 1). Therefore, the various effects of dopamine on the function of stem cells may be a potential target for alleviating the body's inflammatory state. Further study of dopamine and its role in stem cells may provide new ideas for the therapy strategy of inflammatory diseases.

## Data Availability

Not applicable.

## Abbreviations

ESC: Embryonic stem cells
iPSC: Induced pluripotent stem cells
DA: Dopamine
GPCR: G-protein-coupled receptor
SVZ: Subependymal region
CNTF: Ciliary neurotrophic factor
E.V.s: Extracellular vesicles
MSCs: Mesenchymal stromal cells
VEGF: Various pro-angiogenic factors
BMSC: Bone marrow-derived mesenchymal stromal cells
hBMSCs: Human bone marrow mesenchymal stromal cells
GVHD: Graft-versus-host disease
SLE: Systemic lupus erythematosus
LT-HSCs: Long-term hematopoietic stem cells
ST-HSCs: Short-term hematopoietic stem cells
HSPCs: Hematopoietic stem cells and progenitor cells
CMPs: Myeloid progenitors
CLPs: Lymphoid progenitors
TLRs: Toll-like receptors
FEN: Fenoldopam

## References

[1] Warren N, O'Gorman C, Lehn A, Siskind D (2017). Dopamine dysregulation syndrome in Parkinson's disease: a systematic review of published cases. J Neurol Neurosurg Psychiatry.

[2] Goldberg LI (1972). Cardiovascular and renal actions of dopamine: potential clinical applications. Pharmacol Rev.

[3] Marinosci GZ, De Robertis E, De Benedictis G, Piazza O (2012). Dopamine use in intensive care: are we ready to turn it down?. Transl Med UniSa.

[4] Murphy MB (2000). Dopamine: a role in the pathogenesis and treatment of hypertension. J Hum Hypertens.

[5] Feng Y, Lu Y (2021). Immunomodulatory effects of dopamine in inflammatory diseases. Front Immunol.

[6] Regmi S, Pathak S, Kim JO, Yong CS, Jeong JH (2019). Mesenchymal stem cell therapy for the treatment of inflammatory diseases: challenges, opportunities, and future perspectives. Eur J Cell Biol.

[7] Bacakova L, Zarubova J, Travnickova M, Musilkova J, Pajorova J, Slepicka P (2018). Stem cells: their source, potency and use in regenerative therapies with focus on adipose-derived stem cells—a review. Biotechnol Adv.

[8] Kolios G, Moodley Y (2013). Introduction to stem cells and regenerative medicine. Respiration.

[9] Korbling M, Estrov Z (2003). Adult stem cells for tissue repair—a new therapeutic concept?. N Engl J Med.

[10] Xia QP, Cheng ZY, He L (2019). The modulatory role of dopamine receptors in brain neuroinflammation. Int Immunopharmacol.

[11] O'Keeffe GC, Barker RA, Caldwell MA (2009). Dopaminergic modulation of neurogenesis in the subventricular zone of the adult brain. Cell Cycle.

[12] Wang CX, Ge XY, Wang MY, Ma T, Zhang Y, Lin Y (2020). Dopamine D1 receptor-mediated activation of the ERK signaling pathway is involved in the osteogenic differentiation of bone mesenchymal stem cells. Stem Cell Res Ther.

[13] Chen Q, Liu Y, Jeong HW, Stehling M, Dinh VV, Zhou B (2019). Apelin(+) endothelial niche cells control hematopoiesis and mediate vascular regeneration after myeloablative injury. Cell Stem Cell.

[14] Hofland LJ, Feelders RA, de Herder WW, Lamberts SW (2010). Pituitary tumours: the sst/D2 receptors as molecular targets. Mol Cell Endocrinol.

[15] Koga M, Nakao H, Arao M, Sato B, Noma K, Morimoto Y (1987). Demonstration of specific dopamine receptors on human pituitary adenomas. Acta Endocrinol (Copenh).

[16] Cai L, Chen J, Lu J, Li Q, Chen X, Zhang L (2021). Tumor stem-like cells isolated from MMQ cells resist to dopamine agonist treatment. Mol Cell Endocrinol.

[17] Lao CL, Lu CS, Chen JC (2013). Dopamine D3 receptor activation promotes neural stem/progenitor cell proliferation through AKT and ERK1/2 pathways and expands type-B and -C cells in adult subventricular zone. Glia.

[18] Shimazaki T, Shingo T, Weiss S (2001). The ciliary neurotrophic factor/leukemia inhibitory factor/gp130 receptor complex operates in the maintenance of mammalian forebrain neural stem cells. J Neurosci.

[19] Doetsch F, Caille I, Lim DA, Garcia-Verdugo JM, Alvarez-Buylla A (1999). Subventricular zone astrocytes are neural stem cells in the adult mammalian brain. Cell.

[20] Berg DA, Belnoue L, Song H, Simon A (2013). Neurotransmitter-mediated control of neurogenesis in the adult vertebrate brain. Development.

[21] Yang P, Arnold SA, Habas A, Hetman M, Hagg T (2008). Ciliary neurotrophic factor mediates dopamine D2 receptor-induced CNS neurogenesis in adult mice. J Neurosci.

[22] Mori M, Jefferson JJ, Hummel M, Garbe DS (2008). CNTF: a putative link between dopamine D2 receptors and neurogenesis. J Neurosci.

[23] Stockli KA, Lillien LE, Naher-Noe M, Breitfeld G, Hughes RA, Raff MC (1991). Regional distribution, developmental changes, and cellular localization of CNTF-mRNA and protein in the rat brain. J Cell Biol.

[24] Kippin TE, Kapur S, van der Kooy D (2005). Dopamine specifically inhibits forebrain neural stem cell proliferation, suggesting a novel effect of antipsychotic drugs. J Neurosci.

[25] Van Kampen JM, Hagg T, Robertson HA (2004). Induction of neurogenesis in the adult rat subventricular zone and neostriatum following dopamine D3 receptor stimulation. Eur J Neurosci.

[26] Van Kampen JM, Robertson HA (2005). A possible role for dopamine D3 receptor stimulation in the induction of neurogenesis in the adult rat substantia nigra. Neuroscience.

[27] Chen PC, Lao CL, Chen JC (2009). The D(3) dopamine receptor inhibits dopamine release in PC-12/hD3 cells by autoreceptor signaling via PP-2B, CK1, and Cdk-5. J Neurochem.

[28] Sahlgren CM, Pallari HM, He T, Chou YH, Goldman RD, Eriksson JE (2006). A nestin scaffold links Cdk5/p35 signaling to oxidant-induced cell death. EMBO J.

[29] Sood NN (1968). Prevalence of pseudoexfoliation of the lens capsule in India. Acta Ophthalmol (Copenh).

[30] Milosevic J, Schwarz SC, Maisel M, Poppe-Wagner M, Dieterlen MT, Storch A (2007). Dopamine D2/D3 receptor stimulation fails to promote dopaminergic neurogenesis of murine and human midbrain-derived neural precursor cells in vitro. Stem Cells Dev.

[31] Peruzzotti-Jametti L, Bernstock JD, Vicario N, Costa ASH, Kwok CK, Leonardi T (2018). Macrophage-derived extracellular succinate licenses neural stem cells to suppress chronic neuroinflammation. Cell Stem Cell.

[32] Tian T, Cao L, He C, Ye Q, Liang R, You W (2021). Targeted delivery of neural progenitor cell-derived extracellular vesicles for anti-inflammation after cerebral ischemia. Theranostics.

[33] Keating A (2006). Mesenchymal stromal cells. Curr Opin Hematol.

[34] Ding DC, Shyu WC, Lin SZ, Li H (2006). Current concepts in adult stem cell therapy for stroke. Curr Med Chem.

[35] Ding DC, Shyu WC, Lin SZ, Li H (2007). The role of endothelial progenitor cells in ischemic cerebral and heart diseases. Cell Transplant.

[36] Kuo TK, Ho JH, Lee OK (2009). Mesenchymal stem cell therapy for nonmusculoskeletal diseases: emerging applications. Cell Transplant.

[37] Oishi K, Noguchi H, Yukawa H, Hayashi S (2009). Differential ability of somatic stem cells. Cell Transplant.

[38] Cho D-I, Kim MR, Jeong H-Y, Jeong HC, Jeong MH, Yoon SH (2014). Mesenchymal stem cells reciprocally regulate the M1/M2 balance in mouse bone marrow-derived macrophages. Exp Mol Med.

[39] Levoux J, Prola A, Lafuste P, Gervais M, Chevallier N, Koumaiha Z (2021). Platelets facilitate the wound-healing capability of mesenchymal stem cells by mitochondrial transfer and metabolic reprogramming. Cell Metab.

[40] Liu D, Kou X, Chen C, Liu S, Liu Y, Yu W (2018). Circulating apoptotic bodies maintain mesenchymal stem cell homeostasis and ameliorate osteopenia via transferring multiple cellular factors. Cell Res.

[41] Horwitz EM, Le Blanc K, Dominici M, Mueller I, Slaper-Cortenbach I, Marini FC (2005). Clarification of the nomenclature for MSC: The International Society for Cellular Therapy position statement. Cytotherapy.

[42] Renesme L, Pierro M, Cobey KD, Mital R, Nangle K, Shorr R (2022). Definition and characteristics of mesenchymal stromal cells in preclinical and clinical studies: a scoping review. Stem Cells Transl Med.

[43] Tonnesen MG, Feng X, Clark RA (2000). Angiogenesis in wound healing. J Investig Dermatol Symp Proc.

[44] Eming SA, Brachvogel B, Odorisio T, Koch M (2007). Regulation of angiogenesis: wound healing as a model. Prog Histochem Cytochem.

[45] Wu Y, Chen L, Scott PG, Tredget EE (2007). Mesenchymal stem cells enhance wound healing through differentiation and angiogenesis. Stem Cells.

[46] Sasaki M, Abe R, Fujita Y, Ando S, Inokuma D, Shimizu H (2008). Mesenchymal stem cells are recruited into wounded skin and contribute to wound repair by transdifferentiation into multiple skin cell type. J Immunol.

[47] Rustad KC, Wong VW, Sorkin M, Glotzbach JP, Major MR, Rajadas J (2012). Enhancement of mesenchymal stem cell angiogenic capacity and stemness by a biomimetic hydrogel scaffold. Biomaterials.

[48] Kasper G, Dankert N, Tuischer J, Hoeft M, Gaber T, Glaeser JD (2007). Mesenchymal stem cells regulate angiogenesis according to their mechanical environment. Stem Cells.

[49] Shome S, Dasgupta PS, Basu S (2012). Dopamine regulates mobilization of mesenchymal stem cells during wound angiogenesis. PLoS ONE.

[50] Mirones I, Angel Rodriguez-Milla M, Cubillo I, Marinas-Pardo L, de la Cueva T, Zapata A (2014). Dopamine mobilizes mesenchymal progenitor cells through D2-class receptors and their PI3K/AKT pathway. Stem Cells.

[51] Yi SJ, Lee H, Lee J, Lee K, Kim J, Kim Y (2019). Bone remodeling: histone modifications as fate determinants of bone cell differentiation. Int J Mol Sci.

[52] Chamberlain G, Fox J, Ashton B, Middleton J (2007). Concise review: mesenchymal stem cells: their phenotype, differentiation capacity, immunological features, and potential for homing. Stem Cells.

[53] Bystrytska M, Povoroznyuk V, Grygorieva N, Karaban I, Karasevich N (2020). Bone mineral density and risk of osteoporotic fractures in women with Parkinson's disease. J Osteoporos.

[54] Muhlenfeld N, Sohling N, Marzi I, Pieper M, Paule E, Reif PS (2021). Fractures in Parkinson's Disease: injury patterns, hospitalization, and therapeutic aspects. Eur J Trauma Emerg Surg.

[55] Stubbs B, Gaughran F, Mitchell AJ, De Hert M, Farmer R, Soundy A (2015). Schizophrenia and the risk of fractures: a systematic review and comparative meta-analysis. Gen Hosp Psychiatry.

[56] Zhang L, Liu L, Thompson R, Chan C (2014). CREB modulates calcium signaling in cAMP-induced bone marrow stromal cells (BMSCs). Cell Calcium.

[57] Berridge MJ, Lipp P, Bootman MD (2000). The versatility and universality of calcium signalling. Nat Rev Mol Cell Biol.

[58] Kuang Z, Chen Z, Tu S, Mai Z, Chen L, Kang X (2022). Dopamine suppresses osteogenic differentiation of rat bone marrow-derived mesenchymal stem cells via AKT/GSK-3beta/beta-catenin signaling pathway. Stem Cells Int.

[59] Paulus W, Trenkwalder C (2006). Less is more: pathophysiology of dopaminergic-therapy-related augmentation in restless legs syndrome. Lancet Neurol.

[60] Missale C, Nash SR, Robinson SW, Jaber M, Caron MG (1998). Dopamine receptors: from structure to function. Physiol Rev.

[61] Clemens S, Belin-Rauscent A, Simmers J, Combes D (2012). Opposing modulatory effects of D1- and D2-like receptor activation on a spinal central pattern generator. J Neurophysiol.

[62] Ren G, Zhang L, Zhao X, Xu G, Zhang Y, Roberts AI (2008). Mesenchymal stem cell-mediated immunosuppression occurs via concerted action of chemokines and nitric oxide. Cell Stem Cell.

[63] Sun L, Akiyama K, Zhang H, Yamaza T, Hou Y, Zhao S (2009). Mesenchymal stem cell transplantation reverses multiorgan dysfunction in systemic lupus erythematosus mice and humans. Stem Cells.

[64] Zappia E, Casazza S, Pedemonte E, Benvenuto F, Bonanni I, Gerdoni E (2005). Mesenchymal stem cells ameliorate experimental autoimmune encephalomyelitis inducing T-cell anergy. Blood.

[65] Ortiz LA, Dutreil M, Fattman C, Pandey AC, Torres G, Go K (2007). Interleukin 1 receptor antagonist mediates the antiinflammatory and antifibrotic effect of mesenchymal stem cells during lung injury. Proc Natl Acad Sci U S A.

[66] Ma S, Xie N, Li W, Yuan B, Shi Y, Wang Y (2014). Immunobiology of mesenchymal stem cells. Cell Death Differ.

[67] Cao W, Yang Y, Wang Z, Liu A, Fang L, Wu F (2011). Leukemia inhibitory factor inhibits T helper 17 cell differentiation and confers treatment effects of neural progenitor cell therapy in autoimmune disease. Immunity.

[68] Benkhoucha M, Molnarfi N, Dunand-Sauthier I, Merkler D, Schneiter G, Bruscoli S (2014). Hepatocyte growth factor limits autoimmune neuroinflammation via glucocorticoid-induced leucine zipper expression in dendritic cells. J Immunol.

[69] Maffioli E, Nonnis S, Angioni R, Santagata F, Cali B, Zanotti L (2017). Proteomic analysis of the secretome of human bone marrow-derived mesenchymal stem cells primed by pro-inflammatory cytokines. J Proteomics.

[70] Nemoto Y, Kanai T, Takahara M, Oshima S, Nakamura T, Okamoto R (2013). Bone marrow-mesenchymal stem cells are a major source of interleukin-7 and sustain colitis by forming the niche for colitogenic CD4 memory T cells. Gut.

[71] Zou X, Zhang G, Cheng Z, Yin D, Du T, Ju G (2014). Microvesicles derived from human Wharton's Jelly mesenchymal stromal cells ameliorate renal ischemia-reperfusion injury in rats by suppressing CX3CL1. Stem Cell Res Ther.

[72] Shi Y, Wang Y, Li Q, Liu K, Hou J, Shao C (2018). Immunoregulatory mechanisms of mesenchymal stem and stromal cells in inflammatory diseases. Nat Rev Nephrol.

[73] Su J, Chen X, Huang Y, Li W, Li J, Cao K (2014). Phylogenetic distinction of iNOS and IDO function in mesenchymal stem cell-mediated immunosuppression in mammalian species. Cell Death Differ.

[74] Li Y, Qiu W, Zhang L, Fung J, Lin F (2016). Painting factor H onto mesenchymal stem cells protects the cells from complement- and neutrophil-mediated damage. Biomaterials.

[75] Galleu A, Riffo-Vasquez Y, Trento C, Lomas C, Dolcetti L, Cheung TS (2017). Apoptosis in mesenchymal stromal cells induces in vivo recipient-mediated immunomodulation. Sci Transl Med..

[76] King KY, Goodell MA (2011). Inflammatory modulation of HSCs: viewing the HSC as a foundation for the immune response. Nat Rev Immunol.

[77] Liu Y, Chen Q, Jeong HW, Han D, Fabian J, Drexler HCA (2021). Dopamine signaling regulates hematopoietic stem and progenitor cell function. Blood.

[78] Kapoor A, Padmavathi A, Madhwal S, Mukherjee T (2022). Dual control of dopamine in Drosophila myeloid-like progenitor cell proliferation and regulation of lymph gland growth. EMBO Rep.

[79] Agarwala S, Kim KY, Phan S, Ju S, Kong YE, Castillon GA (2022). Defining the ultrastructure of the hematopoietic stem cell niche by correlative light and electron microscopy. Elife.

[80] Sarkar S, Imam SZ, Walters JL, Slikker W, Paule MG, Wang C (2018). Chapter 15—ontogeny of monoamine neurotransmitters. Handbook of developmental neurotoxicology.

[81] Eaves CJ (2015). Hematopoietic stem cells: concepts, definitions, and the new reality. Blood.

[82] Perry JM, Li L (2010). Functional assays for hematopoietic stem cell self-renewal. Methods Mol Biol.

[83] Babovic S, Eaves CJ (2014). Hierarchical organization of fetal and adult hematopoietic stem cells. Exp Cell Res.

[84] Zhang Y, Gao S, Xia J, Liu F (2018). Hematopoietic hierarchy—an updated roadmap. Trends Cell Biol.

[85] Cronkite DA, Strutt TM (2018). The regulation of inflammation by innate and adaptive lymphocytes. J Immunol Res.

[86] Manz MG, Boettcher S (2014). Emergency granulopoiesis. Nat Rev Immunol.

[87] Ueda Y, Cain DW, Kuraoka M, Kondo M, Kelsoe G (2009). IL-1R type I-dependent hemopoietic stem cell proliferation is necessary for inflammatory granulopoiesis and reactive neutrophilia. J Immunol.

[88] Nagai Y, Garrett KP, Ohta S, Bahrun U, Kouro T, Akira S (2006). Toll-like receptors on hematopoietic progenitor cells stimulate innate immune system replenishment. Immunity.

[89] Liu A, Wang Y, Ding Y, Baez I, Payne KJ, Borghesi L (2015). cutting edge: hematopoietic stem cell expansion and common lymphoid progenitor depletion require hematopoietic-derived, cell-autonomous TLR4 in a model of chronic endotoxin. J Immunol.

[90] Takizawa H, Regoes RR, Boddupalli CS, Bonhoeffer S, Manz MG (2011). Dynamic variation in cycling of hematopoietic stem cells in steady state and inflammation. J Exp Med.

[91] Hirai H, Zhang P, Dayaram T, Hetherington CJ, Mizuno S, Imanishi J (2006). C/EBPbeta is required for 'emergency' granulopoiesis. Nat Immunol.

[92] Pietras EM, Mirantes-Barbeito C, Fong S, Loeffler D, Kovtonyuk LV, Zhang S (2016). Chronic interleukin-1 exposure drives haematopoietic stem cells towards precocious myeloid differentiation at the expense of self-renewal. Nat Cell Biol.

[93] Lacoste B, Raymond VA, Cassim S, Lapierre P, Bilodeau M (2017). Highly tumorigenic hepatocellular carcinoma cell line with cancer stem cell-like properties. PLoS ONE.

[94] Clarke MF, Dick JE, Dirks PB, Eaves CJ, Jamieson CH, Jones DL (2006). Cancer stem cells–perspectives on current status and future directions: AACR Workshop on cancer stem cells. Cancer Res.

[95] Yang L, Yao Y, Yong L, Feng Y, Su H, Yao Q (2019). Dopamine D(1) receptor agonists inhibit lung metastasis of breast cancer reducing cancer stemness. Eur J Pharmacol.

[96] Adorno-Cruz V, Kibria G, Liu X, Doherty M, Junk DJ, Guan D (2015). Cancer stem cells: targeting the roots of cancer, seeds of metastasis, and sources of therapy resistance. Cancer Res.

[97] Roney MSI, Park SK (2018). Antipsychotic dopamine receptor antagonists, cancer, and cancer stem cells. Arch Pharm Res.

[98] Yue H, Huang D, Qin L, Zheng Z, Hua L, Wang G (2016). Targeting lung cancer stem cells with antipsychological drug thioridazine. Biomed Res Int.

[99] Wang S, Mou Z, Ma Y, Li J, Li J, Ji X (2015). Dopamine enhances the response of sunitinib in the treatment of drug-resistant breast cancer: Involvement of eradicating cancer stem-like cells. Biochem Pharmacol.

[100] Wang X, Wang ZB, Luo C, Mao XY, Li X, Yin JY (2019). The prospective value of dopamine receptors on bio-behavior of tumor. J Cancer.

[101] Sachlos E, Risueno RM, Laronde S, Shapovalova Z, Lee JH, Russell J (2012). Identification of drugs including a dopamine receptor antagonist that selectively target cancer stem cells. Cell.

[102] Borcherding DC, Tong W, Hugo ER, Barnard DF, Fox S, LaSance K (2016). Expression and therapeutic targeting of dopamine receptor-1 (D1R) in breast cancer. Oncogene.

[103] Dolma S, Selvadurai HJ, Lan X, Lee L, Kushida M, Voisin V (2016). Inhibition of dopamine receptor D4 impedes autophagic flux, proliferation, and survival of glioblastoma stem cells. Cancer Cell.

[104] Kim Y, Kim E, Wu Q, Guryanova O, Hitomi M, Lathia JD (2012). Platelet-derived growth factor receptors differentially inform intertumoral and intratumoral heterogeneity. Genes Dev.

[105] Suzuki S, Okada M, Kuramoto K, Takeda H, Sakaki H, Watarai H (2016). Aripiprazole, an antipsychotic and partial dopamine agonist, inhibits cancer stem cells and reverses chemoresistance. Anticancer Res.

[106] Zhang Y, Wang X (2020). Targeting the Wnt/beta-catenin signaling pathway in cancer. J Hematol Oncol.

[107] Pan B, Huang XF, Deng C (2016). Aripiprazole and haloperidol activate GSK3beta-dependent signalling pathway differentially in various brain regions of rats. Int J Mol Sci.

[108] Singh N, Krishnakumar S, Kanwar RK, Cheung CH, Kanwar JR (2015). Clinical aspects for survivin: a crucial molecule for targeting drug-resistant cancers. Drug Discov Today.

[109] Chinchar E, Makey KL, Gibson J, Chen F, Cole SA, Megason GC (2014). Sunitinib significantly suppresses the proliferation, migration, apoptosis resistance, tumor angiogenesis and growth of triple-negative breast cancers but increases breast cancer stem cells. Vasc Cell.

[110] Campisi J, di Fagagna FD (2007). Cellular senescence: when bad things happen to good cells. Nat Rev Mol Cell Biol.

[111] Paul R, Dorsey JF, Fan Y (2022). Cell plasticity, senescence, and quiescence in cancer stem cells: Biological and therapeutic implications. Pharmacol Ther.

[112] Lopes-Paciencia S, Saint-Germain E, Rowell MC, Ruiz AF, Kalegari P, Ferbeyre G (2019). The senescence-associated secretory phenotype and its regulation. Cytokine.

[113] Bavik C, Coleman I, Dean JP, Knudsen B, Plymate S, Nelson PS (2006). The gene expression program of prostate fibroblast senescence modulates neoplastic epithelial cell proliferation through paracrine mechanisms. Can Res.

[114] Lee YI, Choi S, Roh WS, Lee JH, Kim T-G (2021). Cellular senescence and inflammaging in the skin microenvironment. Int J Mol Sci.

[115] Coppe JP, Desprez PY, Krtolica A, Campisi J (2010). The senescence-associated secretory phenotype: the dark side of tumor suppression. Annu Rev Pathol Mech Dis.

[116] Riessland M, Kolisnyk B, Kim TW, Cheng J, Ni J, Pearson JA (2019). Loss of SATB1 induces p21-dependent cellular senescence in post-mitotic dopaminergic neurons. Cell Stem Cell.

[117] Calcinotto A, Kohli J, Zagato E, Pellegrini L, Demaria M, Alimonti A (2019). Cellular senescence: aging, cancer and injury. Physiol Rev.

[118] Bektas A, Schurman SH, Sen R, Ferrucci L (2018). Aging, inflammation and the environment. Exp Gerontol.

[119] Villar-Cheda B, Dominguez-Meijide A, Valenzuela R, Granado N, Moratalla R, Labandeira-Garcia JL (2014). Aging-related dysregulation of dopamine and angiotensin receptor interaction. Neurobiol Aging.

[120] Jiang Z, Wang J, Sun G, Feng M (2022). BDNF-modified human umbilical cord mesenchymal stem cells-derived dopaminergic-like neurons improve rotation behavior of Parkinson's disease rats through neuroprotection and anti-neuroinflammation. Mol Cell Neurosci.

[121] Wu Y, Hu Y, Wang B, Li S, Ma C, Liu X (2020). Dopamine uses the DRD5-ARRB2-PP2A signaling axis to block the TRAF6-mediated NF-kappa B pathway and suppress systemic inflammation. Mol Cell.

[122] Tan Y, Sun R, Liu L, Yang D, Xiang Q, Li L (2021). Tumor suppressor DRD2 facilitates M1 macrophages and restricts NF-kappa B signaling to trigger pyroptosis in breast cancer. Theranostics.

[123] Moiseeva O, Deschenes-Simard X, St-Germain E, Igelmann S, Huot G, Cadar AE (2013). Metformin inhibits the senescence-associated secretory phenotype by interfering with IKK/NF-B activation. Aging Cell.

[124] Luo Y, Roth GS (2000). The roles of dopamine oxidative stress and dopamine receptor signaling in aging and age-related neurodegeneration. Antioxid Redox Signal.

